# Coping Strategies and Perception of Patients With Breast Cancer Post‐Surgery in an Underserved Region of Nigeria

**DOI:** 10.1002/pon.70370

**Published:** 2025-12-30

**Authors:** Ayodeji A. Bioku, Bonnie Lu, Paige Harris, Foluke O. Sarimiye, Jummai D. Jimeta‐Tuko, Navya Manoj, Tinuke O. Olagunju, Olubukola O. Kolawole, Abdullah H. Alqahtani, Britta K. Ostermeyer, Andrew T. Olagunju

**Affiliations:** ^1^ Federal Medical Centre Birnin Kebbi Nigeria; ^2^ Department of Psychiatry The Royal Hobart Hospital Hobart Tasmania Australia; ^3^ Michael G. Degroote School of Medicine McMaster University Hamilton Ontario Canada; ^4^ Department of Psychiatry and Behavioral Neurosciences McMaster University Hamilton Ontario Canada; ^5^ Forensic Psychiatry Program St. Joseph's Healthcare Hamilton Hamilton Ontario Canada; ^6^ Department of Radiation Oncology University of Ibadan Ibadan Nigeria; ^7^ Oncology Unit Medical Services Department Diagnostic and Treatment Center, Central Bank of Nigeria Abuja Nigeria; ^8^ Public Health Agency of Canada Toronto Ontario Canada; ^9^ Psychiatry Division Medicine Department Johns Hopkins Aramco Healthcare (JHAH) Dhahran Saudi Arabia; ^10^ Department of Psychiatry and Behavioral Sciences The University of Oklahoma College of Medicine Oklahoma City Oklahoma USA; ^11^ Federal Neuropsychiatric Hospital Calabar Nigeria; ^12^ Discipline of Psychiatry University of Adelaide Adelaide Australia

**Keywords:** breast cancer, coping strategies, distress, mastectomy, quality of life, surgery

## Abstract

**Background:**

Patients' coping styles, perceptions, and attitudes are important for healthy living and survivorship in breast cancer.

**Aim:**

To assess factors associated with coping styles in patients with breast cancer surgery in an underserved population in Northern Nigeria.

**Methods:**

This cross‐sectional study included 72 patients with breast cancer post‐surgery. Data was collected on clinico‐demographic variables, patients' perceptions, the World Health Organization Quality of Life (WHOQOL‐BREF), the General Health Questionnaire (GHQ‐12), and the Coping Strategy Inventory (CSI‐32).

**Results:**

The mean age of participants was 45.9 (±9.1) years. Over 80% of participants underwent mastectomy, with 93.1% reporting complications. Notwithstanding, participants largely perceived that the procedure was life‐saving, and most did not feel the need for a breast prosthesis. Notably, 70.8% reported an increased tendency to withdraw socially, but only 34.7% experienced functional difficulties with daily activities or chores. Patients practiced different engagement‐disengagement coping strategies, albeit use of multiple dimensions of engagement coping was pronounced in a greater fraction (percentile) of patients. Multiple aspects of perception (including a lack of satisfaction with clothes fitting, feelings of incompleteness, greater time since surgery), and the experience of psychological distress were associated with disengagement coping. On the other hand, engagement coping was more likely in patients who were satisfied with clothing fit, experienced less impact on daily living, and had an improved quality of life.

**Conclusion:**

Coping in patients with breast cancer post‐surgery is multifaceted, varying by individual perception and psychosocial wellbeing. Future research is needed to guide interventions that bolster psychosocial well‐being to promote healthy coping.

## 1 Introduction

Breast cancer is associated with a significant global burden, with approximately 2.3 million new diagnoses and 670,000 breast cancer‐related deaths annually [1]. Although incidence rates are higher in high‐income countries, the rising mortality rates, significant burden and decreased survivorship in low‐ and middle‐income countries highlight the need for preventative screening, accessible treatment, and holistic care (defined as a management approach that treats the whole person by considering every aspect—physical, social, psychological, spiritual, and lifestyle—of patients' experiences) to improve survivorship [2, 3, 4, 5, 6]. Nigeria, the most populous country in Africa and the seventh largest in the world by population, has one of the highest age‐matched breast cancer mortality rates globally, and this is compounded by the growing cancer burden [1, 3, 4]. Combined chemotherapy, radiation, and surgery are often recommended to improve survival of breast cancer patients; however, many patients present at later stages of disease, leading to complicated treatments, poorer outcomes, and negative survivorship experiences [5, 6]. Surgery remains a key component of breast cancer treatment worldwide and is the second most prevalent form of treatment sought by breast cancer patients after chemotherapy [6]. A recent study from Nigeria indicated that the majority of breast cancer patients undergoing surgery received modified radical mastectomy (MRM), followed by simple mastectomy and lumpectomy [7]. While partial mastectomy and lumpectomy are considered breast‐conserving, MRM involves the removal of the entire breast and axillary lymph nodes [6, 7].

The diagnosis and surgical treatment of breast cancer can have far reaching ramifications, including major changes in body image and function, lifestyle, worldview, and self‐concept, prompting unique psychosocial responses and burden for patients and their families [8]. Notably, patients' perception of their illness (which encompasses the understanding of their health condition, their beliefs about its cause, symptoms, treatment, consequences, and timeline) and their coping styles are crucial to shaping their survivorship experience [9]. While several factors can shape patient's perception and breast cancer illness experience, emotional distress—defined as a state of emotional suffering with features of anxiety (e.g., worrying restlessness; feeling tense), depression (e.g., loss of interest; sadness; hopelessness), social dysfunction, and negative emotions—remains an important and common factor among patients with breast cancer [10, 11, 12]. Patient distress is often multifactorial (including psychological, cognitive, behavioral, social, and spiritual dimensions), and associated with unpleasant emotional experiences and negative psychosocial response [12, 13]. Moreover, the various aspects of distress can contribute to patients' attitudes and behaviors toward their diagnosis, as well as their survivorship experience [13, 14].

For patients undergoing breast cancer surgery, changes in body image and function can have unique effects on illness perception and their survivorship experience [9, 12, 15]. Body image involves multiple aspects, including feeling feminine/attractive (affective), avoiding people because of appearance (behavioral), and satisfaction with appearance (cognitive) [12, 15]. Notably, there are indications to suggest that women cope better with breast cancer and its treatment when they have a greater conceptualization of their body image [10, 12, 13, 14]. Age constitutes an important factor associated with body image perception, concerns and the wellbeing of patients receiving breast cancer surgical treatment, with younger women being more negatively impacted [10, 15]. Heightened body image concerns, and negative perceptions have been linked to the surgical removal of breast, scaring, and other physical changes among young women, and in turn cause significant deterioration in their quality of life, overall wellbeing, and can result in poor survivorship experience [13, 15, 16]. Other factors like educational level, coping style, relationship satisfaction, cultural beliefs and the information provided prior to treatment about the potential impacts of surgical treatment can impact patient's perception or self‐acceptance and survivorship experience in breast cancer [12, 14, 16, 17].

Notably, coping styles (defined as the thoughts and behaviors mobilized to manage internal and external stressful situations) have been linked with adaptation, tolerance or alleviation of stress responses, psychological adjustment to breast cancer treatment, and healthy survivorship experience [17, 18]. For example, previous studies showed that coping styles involving problem‐solving and emotional expression may promote mental wellbeing, healthy treatment decisions and resiliency in patients with breast cancer during treatment and recovery [18, 19, 20]. However, misconceptions about breast cancer and surgical treatment (particularly regarding the risks and outcomes of surgery) significantly limit treatment, recovery and survivor outcome, albeit prevalent particularly in resource‐restricted settings like Nigeria [5, 6, 7, 21, 22, 23]. These beliefs can delay patient's presentation for treatment, exacerbate psychological distress and hinder their ability to cope effectively with the challenges of treatment 20, 21, 22, 23]. On the other hand, an improvement in wellbeing and quality of life, especially when linked to treatment, can present an important opportunity to correct misconceptions and promote better engagement and healthy coping strategies, although this is yet to be fully explored [6, 15, 17, 18, 19, 20].

There is a significant gap in research concerning unraveling the ramifications of living with breast cancer and its treatment, especially those that addresses coping styles, perception about surgical treatment and survivorship post‐surgery in resource‐restricted contexts. Breast cancer diagnosis and treatment, especially surgeries, have several health, economic, cultural, personal, and psychosocial consequences on individuals and their families, making them public health issues of importance [1, 5, 6, 7, 23, 24, 25]. Understanding patient coping styles, perspectives regarding breast cancer treatment, and experiences after surgery may provide important insight for clinicians as to how best to support patients throughout their treatment (especially before, during and after surgery) to improve their survivorship experiences.

In Nigeria, there has been a little effort to explore the general psychosocial effects of breast cancer treatment [6, 20, 22, 23, 24, 25, 26, 27, 28]; however, there is a notable lack of quantitative research exploring coping strategies, perception and their relationship with psychological distress and quality of life [26]. The present study aims to shed light on the relationship of coping strategies with perceptions, psychological distress and quality of life among patients with breast cancer post‐surgery in an underserved region in Northwestern Nigeria. We postulated that the variance in patient's coping styles will be explained by multiple factors, including quality of life, perceptions, clinico‐demographic attributes, and psychological distress. Ultimately, we hope that findings from this study would extend current knowledge on coping style and the survivorship experience of patients with surgical treatment for breast cancer, supporting evidence‐informed initiatives and further research to bolster resilience and improve survivorship. The specific study objectives are to:

- Assess patients' perceptions of breast cancer surgery as a treatment modality.
- Describe the multiple aspects of coping strategies practiced by patients with breast cancer post‐surgery.
- Assess the relationship of coping strategies (engagement vs. disengagement) with multiple aspects of perception about treatment, quality of life and psychological distress post‐breast cancer surgery.
- Investigate factor that contributes to the variance in engagement‐disengagement coping, discuss the implications of the study findings and highlight relevant recommendations.

## 2 Methods

### 2.1 Study Populations

This cross‐sectional study is derived from an analysis of data collected from 72 breast cancer patients following surgical treatment in an underserved region in North Western Nigeria [27]. The study population consisted of all adults who had undergone breast surgery following a diagnosis of breast cancer at two tertiary hospitals in the North‐Western part of Nigeria: the Federal Teaching Hospital Birnin‐Kebbi and the Usmanu Danfodiyo University Teaching Hospital, Sokoto. These hospitals operate separate oncology departments that offer surgery and other forms of cancer treatment to patients within their catchment areas, covering multiple states in the North‐West and an estimated population of about 13 million people. Oncology services are delivered by a multi‐disciplinary team of professionals, consisting of oncologists with a ratio of 3: 13 million people, residents' doctors (6–13 million), nursing staff (9–13 million), rehabilitation therapists (3–13 million), radiotherapists (2–13 million), radiotherapy technicians (3–13 million), and counsellors (4–13 million). Both physicists and psychologists had a ratio of one to 13 million people. Payment for care and services is largely out of pocket due to poor health insurance coverage [28, 29].

Eligible participants were patients with breast cancer and mastectomy who provided informed consent and were recruited over a 30‐month study period. The subjects were females who had surgeries following a histological diagnosis of breast cancer at least 2 months before the date of data collection. We included adults aged 18 years or older with proficiency in the English language, who were clinically stable enough to engage in an interview. We excluded those with other forms of surgery as well as those who declined consent to participate. A total of 86 patients were approached for recruitment. However, 72 participants (approximately 84% response rate) were recruited into the study.

### 2.2 Study Procedure and Instrument

Ethical approval (ethics number: BK/HP/045/P/517/VOLV/043) was obtained before study enrollment, and all participants provided written informed consent. Following informed consent, participants were administered the study instrument, which is made up of four different components.

The first component of the study instrument consisted of a *study designed questionnaire* to collect basic information on socio‐demographic and illness‐related parameters such as age, marital status, family setting, level of education, occupation, types of surgery, number of surgeries, duration since surgery, presence of chronic medical illness, counseling before surgery, previous diagnosis of mental illness, and complications after surgery. A second part of this questionnaire was used to gather information on the participants' general perceptions regarding breast surgery, including questions on their perceptions about the surgery scar, general feelings about the surgery, general appearance and body image, the need for a prosthesis, and the feeling about the possibility of metastasis.

The second component of the instrument contained the 12‐item version of the *General Health Questionnaire‐(GHQ‐12*) [17] used to elicit psychological distress. The GHQ‐12 was administered to generate scores that were rated on a bimodal scoring scheme (0‐0‐1‐1) to produce total scores that ranged from zero to 12. A higher score indicates greater severity of psychological distress. The instrument has been validated and widely used in Nigeria [17, 27, 30, 31].

The third component of the instrument was the 26‐item *World Health Organization Quality of Life (WHOQoL‐BREF)* [32]. It was administered to measure several dimensions of quality of life (QoL). The WHOQoL has four broad domains that relate to an individual idea of QoL, namely physical health, psychological health, social relationships and environment domains. All the responses are scored on a range of one to five, and the mean score in each domain was calculated and converted to transformed scores according to the procedure manual. A higher score on the scale is positively correlated with a better QoL. The scale is validated and has been well‐used in Nigeria [20, 24].

Lastly, the final component of the instrument collected data regarding coping strategies using the *32‐item Coping Strategy Inventory (CSI‐32)* [33]. The 32–item CSI rates the general frequency with which paticipants utilize each listed coping strategy on the survey, indicating their choice on a five‐point Likert scale in the following manner: 1 = “not at all”, 2 = “a little”, 3 = “somewhat”, 4 = “much” and 5 = “very much”. The computation of the score on each item of the 32‐items CSI can be used to derive eight primary, four secondary, and two tertiary subscales. The primary subscales consist of problem‐solving (derived from combining scores on items 1, 9, 17 and 25), cognitive restructuring (items 2, 10, 18 and 26), expression of emotion (items 3, 11, 19 and 27), social contact (items4, 12, 20 and 28), problem avoidance (items 5, 13, 21, and 29), wishful thinking (items 6, 14, 22 and 30), self‐criticism (items 7, 15, 23 and 31) and social withdrawal (items 8,16, 24 and 32). The secondary subscales are derived by combining the primary subscales and include: problem‐focused engagement (derived from combining problem‐solving and cognitive restructuring), emotion‐focused engagement (derived from social contact and express emotion), problem‐focused disengagement (derived from problem avoidance and wishful thinking), and emotion‐focused disengagement (derived from self‐criticism and social withdrawal). Finally, two tertiary subscales are derived from combining the secondary subscales and include engagement (combining both problem‐focused engagement and emotion‐focused engagement) and disengagement (derived from problem‐focused disengagement and emotion‐focused disengagement). Overall, the score on each item can range from zero to 5, and mean scores are calculated for each subscale. The higher the mean, the more the participants engage in the coping strategies depicted by the subscales. The CSI‐32 has good psychometric properties and has been widely used in Nigeria [20, 33].

### 2.3 Data Analysis

Data analysis was conducted using SPSS Version 22.0. We used descriptive statistics, including frequencies and percentages to present categorical variables. Continuous variables were described by mean with standard deviation and percentiles. An independent sample *t*‐test was performed to assess the relationship of dichotomized clinico‐socio‐demographic variables with engagement versus disengagement coping based on CSI‐32 assessment. Pearson's correlation was performed to test the relationship of different aspects of coping (CSI‐32 subscales) with QoL (WHOQoL‐BREF) and psychological distress (GHQ‐12). We included effect sizes categorized based on Cohen's guideline using Pearson's coefficient (*r*) = (*r* = 0.1 is small; *r* = 0.3 is medium and *r* = 0.5 is large). Regression analysis was conducted with disengagement (model 1) and engagement coping (model 2) as the dependent variables [DV]. The independent variables [IV] including quality of life, psychological distress, satisfaction with clothing fit, feelings of incompleteness and duration since surgery (factors that were significantly associated with disengagement and engagement coping during bivariate analysis) were included in the analysis to identify factors contributing to the variance in disengagement‐engagement coping. A significant *p*‐value was set at 0.05 with a 95% confidence interval (CI).

## 3 Results

### 3.1 Characteristic of Participants and Their Perception About Breast Cancer Surgery

**FIGURE 1:**
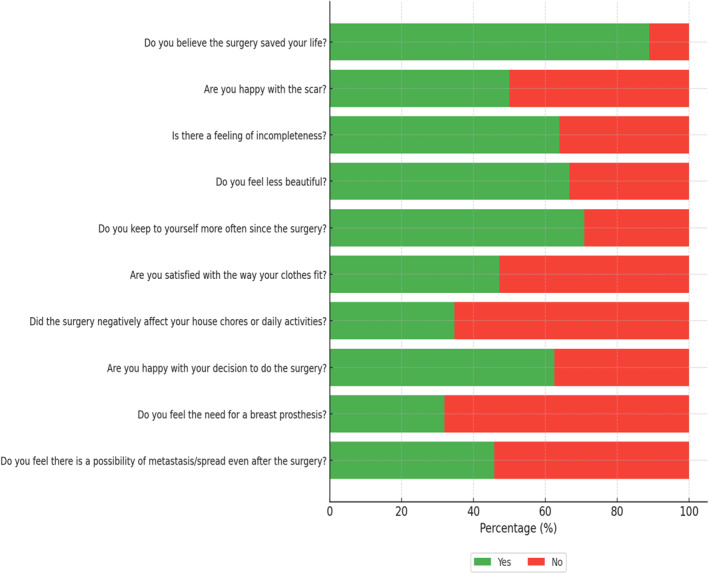
Participants' perceptions about treatment with surgery.

The majority of the participants were in their fifth decade of life and older (76.4%), had a mastectomy (83.3%) and reported surgical complications (93.1%). See Table 1 for additional details. While the majority of the participants (88.9%) believed that surgery saved their life, half (50.0%) were unhappy with the scar, two‐thirds (66.7%) described feeling less beautiful, and 63.9% reported a sense of incompleteness. Additionally, 52.8% were dissatisfied with the fit of their clothes. Despite these concerns, most participants (68.1%) did not feel the need for a breast prosthesis. The surgery also impacted social behavior, with 70.8% reporting increased tendencies to keep to themselves; however, only one‐third (34.7%) indicated difficulties with daily activities or chores. Uncertainty about the possible presence of metastasis remained a concern for just under half (45.8%) of the participants. See Figure 1 for additional details.

**TABLE 1:** Characteristics of the study participants (n = 72).

Variables	Frequency (*n*)	Percentage (%)
Age		
40 years or less	17	23.6
41 years and above	55	76.4
Education		
Primary or less	37	51.4
Secondary	8	11.1
Post‐secondary	27	37.5
Occupation		
Unemployed	38	52.8
Petty trading/Farming	16	22.2
Employed	18	25.0
Marital Status		
Single	4	5.6
Married	42	58.3
Separated/divorced	3	4.2
Widowed	23	31.9
Religion		
Islam	58	80.6
Christianity	14	19.4
Ethnicity		
Hausa	48	66.7
Yoruba	8	11.1
Igbo And others	16	22. 2
Family/Marriage Setting		
Polygamous	38	52.8
Monogamous	34	47.2
Living Circumstances		
Living with husband	38	52.8
Living with relatives(s)	23	31.9
Living with parent(s)	5	6.9
Living alone	6	8.3
Type of Surgery		
Lumpectomy	12	16.7
Mastectomy	60	83.3

Abbreviations: n = number of subjects; % = percentage.

### 3.2 Dimensions of Coping Strategies Practiced by Participants

Table 2 presents the findings on different dimensions of coping strategies practiced by the participants. The mean (±SD) of participants' CSI‐32 scores in the primary subscales ranged from 12.97 ± 3.07 (expression of emotions coping) to 8.91 ± 4.55 (self‐criticism). Considering the percentiles, half of the participants' scored ≤ 10 (50^th^ percentile) for problem‐solving and 13 at 75th percentile. Cognitive restructuring scores improved from 12 to 15 at the 50th and 75th percentiles, respectively. Participants used social support moderately, with the score of 14 at the 75th percentile, indicating that a good portion of participants lean on others. Many participants place significant value on emotional expression, with a relatively high score (11) at 25^th^ percentile and there was progressive increase in the scores at the 50th (13) and 75th (15.8) percentiles. Interestingly, there seems to be clustering of participants in the middle for avoidance, as both the 50th and 75th percentiles scores are 14. Wishful thinking shows a wide spread with a score of 14 at the 50th percentile and 16.75 at the 75th. At the 25th percentile, self‐criticism is quite low (4), meaning many participants rarely engage in self‐critical thought but increased appreciably to 12 at 75th percentile. The score of 10 at 50th percentiles suggests a moderate tendency to withdraw socially.

**TABLE 2:** Distribution of participants scores across different aspects of coping (CSI‐32 subscales).

Variables	Mean	SD	95% CI	25^th^ percentile	50^th^ percentile	75^th^ percentile
Primary subscales						
Problem‐solving	10.15	3.23	9.39–10.91	8.00	10.00	13.00
Cognitive restructuring	12.11	3.59	11.27–13.01	9.00	12.00	15.00
Express emotions	12.97	3.07	11.25–13.69	11.00	13.00	15.75
Social support	11.67	2.93	10.98–12.35	9.00	11.00	14.00
Problem avoidance	12.45	3.33	11.68–13.24	10.00	14.00	14.00
Wishful thinking	12.87	4.88	11.73–14.02	10.00	14.00	16.75
Self‐criticism	8.91	4.55	7.85–9.99	4.00	8.00	12.00
Social withdrawal	9.48	3.47	8.67–10.30	7.00	10.00	11.00
Secondary subscales						
Problem focusing	22.56	6.23	21.10–24.03	17.00	22.00	28.00
Emotion focusing	24.63	4.76	23.52–25.76	21.00	25.00	27.00
Problem‐focused engagement	25.33	6.68	23.76–26.90	20.00	25.00	32.00
Emotion‐focused disengagement	18.40	5.92	17.01–19.79	14.00	16.50	22.00
Tertiary subscales						
Engagement	46.94	9.69	44.67–49.22	38.00	48.50	54.00
Disengagement	43.73	9.98	41.39–46.08	37.00	46.00	51.00

Abbreviations: CI‐confidence interval; SD−standard deviation.

Findings on the secondary CSI‐32 subscales showed a similar pattern, improving from the 50^th^ to 75^th^ percentiles for all subscales, including problem focusing (17–28), emotion focusing (21–27), problem‐focused engagement (20–32) and emotion‐focused disengagement (14–22).

Engagement coping (*M* = 46.94, SD = 9.69) showed a better mean score compared to disengagement coping (*M* = 43.73, SD = 9.98). Similarly, engagement coping maintained a better score at the 50th (48.5) and 75^th^ (54) percentiles compared to disengagement coping with 46 at the 50^th^ and 51 at the 75^th^ percentiles.

Overall, the study results indicate that participants seem to lean more heavily on engagement coping strategies (including problem‐solving, cognitive restructuring, and expressing emotions) compared to disengagement strategies like self‐criticism or social withdrawal, albeit there was a degree of variation, particularly for wishful thinking and problem avoidance.

### 3.3 Relationship of Engagement/Disengagement Coping With Clinico‐Demographic Variables and Perception

Supplementary documents 1 and 2 showed findings on the relationships of engagement and disengagement coping with clinico‐demographic characteristics. The statistical analysis showed no significant association between engagement‐disengagement coping and any of the measured clinic‐demographic variables. On the other hand, some aspects of perception were observed to have a statistically significant association with disengagement coping style, including dissatisfaction with clothing fitness (*F* = 3.89; MD = −5.13 *t*(70) = −2.24, *p* = 0.028), feeling of incompleteness (*F* = 0.19; MD = −6.27 *t*(70) = 2.67, *p* = 0.0009) and duration since surgery (*F* = 0.2; MD = −5.06; *t*(70) = −1.19, *p* = 0.032). Engagement coping was associated with the duration since‐surgery (*F* = 1.53; MD = 2.522; *t*(70) = 0.40; −1.049; *p* = 0.038), feelings of incompleteness (*F* = 0.67; MD = 3.39; *t*(70) = 0.402; *p* = 0.019), satisfaction with clothing (*F* = 3.55; MD = −2.27; *t*(70) = ‐1.23; *p* = 0.025), and impact on daily living (*F* = 0.265; MD = 2.17; *t*(70) = 0.903; *p* = 0.037).

### 3.4 Correlation of Dimensions of Coping With Quality of Life and Psychological Distress

As shown in Table 3, Several dimensions of coping styles demonstrated significant correlation with psychological distress (GHQ‐12 scores) and quality of life (WHO‐BREF scores). We found a negative correlation between cognitive restructuring (*r* = −0.28, *p* = 0.019, small effect size), problem avoidance (*r* = −0.25, *p* = 0.031, small effect size) and psychological distress. Conversely, positive correlations were observed between wishful thinking (*r* = 0.32, *p* = 0.006, medium effect size), social withdrawal (*r* = 0.61, *p* < 0.001, large effect size) and psychological distress. Also, emotion‐focused disengagement (*r* = 0.49, *p* < 0.001, medium effect size) and disengagement coping (*r* = 0.36, *p* = 0.002, medium effect size) were significantly correlated with psychological distress.

**TABLE 3:** Correlation of different dimensions of coping with psychological distress and quality of life.

Coping dimensions (CSI‐32)	Psychological distress		Quality of life	
	r	*p*‐value	r	*p*‐value
Primary subscales				
Problem solving	−0.29	0.078	0.22	0.063
Cognitive restructuring	−0.28	**0.019**	0.31	**0.009**
Express emotions	−0.10	0.385	0.13	0.281
Social support	0.05	0.691	0.11	0.359
Problem avoidance	−0.25	**0.031**	0.20	0.094
Wishful thinking	0.32	**0.006**	0.01	0.945
Self‐criticism	0.16	0.168	−0.03	0.801
Social withdrawal	0.61	**< 0.001**	−0.34	**0.003**
Secondary subscales				
Problem focusing engagement	0.30	**0.011**	0.17	0.149
Emotional focusing engagement	−0.04	0.752	0.15	0.379
Problem focusing disengagement	0.11	0.370	0.11	0.379
Emotional focusing disengagement	0.49	**< 0.001**	−0.23	0.058
Tertiary subscales				
Engagement	−0.19	0.103	0.25	**0.003**
Disengagement	0.36	**0.002**	−0.06	0.601

*Note: p*‐values in bold indicate statistical significance at *p* < 0.05, effect size categorization based on Cohen's guidelines of Pearson's coefficient [*r*] = (*r* = 0.1 is small; *r* = 0.3 is medium and *r* = 0.5 is large).

On the other hand, cognitive restructuring (*r* = 0.31, *p* = 0.009, medium effect size), social withdrawal (*r* = −0.34, *p* = 0.003, medium effect size) engagement coping (*r* = 0.25, *p* = 0.003, small effect size) were significantly correlated with quality of life.

### 3.5 Regression Analysis of Factors Associated With Coping With Disengagement and Engagement Coping

As presented in Table 4, a total of 45.2% of the variance in disengagement coping was explained in the model 1. There was a statistically significant association of disengagement coping with the lack of satisfaction with clothes fittings post‐operatively (*p* < 0.001), feelings of incompleteness (*p* = 0.004), longer duration since surgery (*p* = 0.014), and the experience of psychological distress (*p* < 0.001), contributing to its variance. On the other hand, 25.4% of the variance in engagement coping was explained in Model 2, showing a significant association of engagement coping with satisfaction with clothing fit (*p* = 0.046), less impact on daily living (*p* = 0.040) and improved quality of life (*p* = 0.02).

**TABLE 4:** Regression analysis of factors associated with disengagement and engagement coping.

Variables						95% CI	
	*R* ^2^	B	SE	Beta	*p*	Lower	Upper
Disengagement [DV]	0.452						
Satisfaction with clothing fit^a^		−7.92	2.08	−0.40	**< 0.001**	**3.756**	**12.076**
Feelings of incompleteness		−6.15	2.18	−0.32	**0.004**	**−10.866**	**−2.161**
Duration since surgery		5.47	2.17	0.25	**0.014**	**1.127**	**9.811**
Psychological distress		9.89	2.18	0.48	**< 0.001**	**5.635**	**14.344**
QOL		−0.19	0.101	−0.26	0.144	−0.052	0.351
(Constant)		5.84	10.10		0.565	−14.356	26.030
Engagement [DV]	0.254						
Satisfaction with clothing fit^a^		4.84	2.37	0.25	0.046	0.090	9.584
Impact on daily living^b^		−5.99	2.86	−0.29	**0.040**	**−11.703**	**−0.278**
Feelings of incompleteness^c^		−2.21	2.49	−0.11	0.377	−7.177	2.757
Duration since surgery		1.88	2.48	0.09	0.450	−3.070	6.838
QOL		0.27	0.12	0.49	**0.020**	**0.044**	**0.504**
(Constant)		31.47	11.52		0.008	8.430	54.512

*Note:* *p*‐values in bold indicate statistical significance at *p* < 0.05.

Abbreviations: B = Unstandardized regression coefficient; Beta = Standardized regression coefficient; CSI = Coping strategies inventory; 95% CI = 95% Confidence interval; df = Degree of freedom; QOL = Quality of Life; *R* ^2^ = Coefficient of determination; SE = Standard error; t = Mean square.

^a^ Are you satisfied with the way your clothes fit?

^b^ Did the surgery negatively affects your house chores or daily activities?

^c^ Is there a feeling of incompleteness?

## 4 Discussion

Surgery, especially mastectomy, is a common treatment modality to stem the burden of breast cancer and improve survivorship, albeit has several major ramifications on psychosocial wellbeing [1, 4, 6]. Coping styles are thought to be key issues in the survivorship experiences of patients with breast cancer pre and post treatment [34], although little is known about the factors associated with coping styles, especially in resource restricted settings. This study highlights findings on coping styles and perception among patients who had breast surgery in an underserved region, offering insight into the factors associated with engagement‐disengagement coping in patients with breast cancer following surgery.

### 4.1 Perception of Participants About Breast Cancer Surgery as a Treatment

The majority of participants reported feelings of incompleteness, reduced self‐esteem, dissatisfaction with surgical scars, and increased social isolation following breast cancer surgery. These findings are worthy of highlighting, albeit consistent with previous literature which highlights negative perceptions about physical appearance in patients with mastectomy, characterized by a shift toward clothing that conceals the absence of a breast, and a diminished sense of self [7, 34, 35]. Such perceptions are particularly relevant in underserved contexts, where limited access to reconstructive surgery is driven by prohibitive cost, and a lack of resources and expertise can exacerbate concerns about body image and identity [21, 28, 34]. This problem and delay to access treatment is further compounded by misconceptions and fear of mastectomy, driven by societal and personal stigma. For instance, a study conducted in a comparable context in Ghana found that confirmed breast cancer patients delayed treatment by an average of 1 year due to fears surrounding the perceived impacts of mastectomy on marriage, womanhood, and attractiveness, as well as misconceptions about surgical outcomes, including mortality [21]. These findings stress an urgency for comprehensive patient education to dispel misconceptions, reduce treatment delays and promote positive survivorship experience.

Most participants in this study did not report significant challenges with daily activities or household chores following surgery. Previous observational studies have indicated good recovery and a return to activities of daily living (ADL) among patients who underwent breast cancer surgery, although they highlighted variability in the time taken for functional recovery and the return to ADL, depending on the type of surgery (mastectomy with or without immediate reconstruction) and post‐surgery complications such as pain [6, 7, 8]. A modified plan for returning to ADL, along with an effective integration of a healthy lifestyle and an individually tailored exercise regimen are recommended approaches for functional recovery after breast cancer surgery [8].

The desire for breast prostheses was limited in participants in our study. Mixed attitudes toward prostheses among post‐mastectomy patients have been observed in previous studies [7, 35]. While some participants noted reluctance due to perceived discomfort or concerns regarding health effects, others expressed a preference for breast‐conserving surgery or reconstruction [7, 35]. Support systems, involving family, local institutions, testimonials from mastectomy survivors, and faith‐based as well as culturally‐embedded support mechanisms have been shown to play a crucial role in addressing misperception about breast cancer surgery/prosthesis, promoting acceptance of breast cancer surgery [7, 8, 35].

### 4.2 Dimensions of Coping Strategies Practiced by Participants

Findings in our study indicates a greater reliance on engagement coping compared to disengagement coping. These findings underscore both similarities and differences when compared with the observations in previous studies of chronic conditions, where coping strategies play significant roles in illness progression and psychological outcomes [20, 36]. Similar to our study, existing research across different chronic conditions and cancers suggests a diverse range of coping strategies utilized by participants, and that these coping styles are multidimensional and the observed variations mixed as well as related to environmental, systemic, cultural, personal, or disease‐specific factors among others [36, 37, 38, 39].

### 4.3 Factors Associated With Engagement and Disengagement Coping

Body image concerns such as dissatisfaction with clothing fit (observed in 52.8% of our cohorts), feelings of incompleteness, an extended time since surgery, and the presence of psychological distress were significantly associated with disengagement coping. Body image concerns—often altered by breast cancer surgery—represent one of the most significant distressing challenges in breast cancer patients [26, 34]. Hence, our study finding is not surprising and align with previous studies [38] that noted that passive and less active coping strategies such as disengagement tend to correlate with body image issues or concerns [35, 36, 37, 38], albeit more research is needed to understand how these dynamics profile across patient subgroups and prospectively in breast cancer survivors.

Another interesting finding is that participants with breast cancer surgery with improved quality of life practiced engagement coping while individuals with psychological distress were more likely to report disengagement coping. Prior studies have linked active coping styles and better engagement with higher quality of life in breast cancer patients [38, 39]. It is possible that major positive impacts on daily life and wellbeing, such as improvement in performing routine activities and independence with less reliance on others, may encourage active‐engagement coping, especially if patients attribute these improved outcomes to the surgical treatment.

### 4.4 Study Limitations

This study has several limitations that warrant consideration. First, the study population comprised of a relatively small sample drawn exclusively from two tertiary hospitals, which may limit the generalizability of the findings to a broader population of people with breast cancer. Second, reliance on self‐reported measures introduces the potential for response bias, which may not fully reflect the complex interactions between psychosocial, and behavioral factors influencing coping strategies. That said, integrating patients' perspectives or subjective evaluations of their experiences remains sacrosanct in psychosocial oncology services. Third, the cross‐sectional design limits the ability to establish causal relationships or examine changes in coping strategies and other parameters over time. Moreover, the coping strategies assessed post‐surgery may not be those employed solely during or immediately after treatment. In light of these limitations, future research should use robust sample size, incorporate longitudinal measures to better understand the temporal dynamics and causal pathways underlying the identified associations and cover a broader context in Nigeria or sub‐Saharan Africa. Additionally, it is important that future research use validated measures and conduct multimodal assessments—combining self‐reports with objective measures—to provide a more comprehensive and accurate evaluation of the factors influencing coping strategies among breast cancer patients. The use of experience sampling methodology [40] to study coping strategy in this population in future studies may confer some advantages.

### 4.5 Implications and Recommendations

The study's findings suggest the potential benefits of holistic management and improved wellbeing in patients with breast cancer surgery, particularly because as quality of life improves, patients are more likely to show a higher engagement and lower disengagement coping. Also, different aspects of patient's perception about breast cancer surgery seem to be crucial for promoting engagement coping. Hence our study findings buttressed the potential beneficial effects of promoting interventions to enhance quality of life‐wellbeing into the care of patients with breast cancer to improve coping. Popular guidelines recommend effective integration of universal, targeted and specialized psycho‐social support to improve wellbeing and quality of life.

On another note, personalized psychoeducation targeting myths, misconceptions and unhealthy perceptions can enhance engagement coping and in turn promote a positive survivorship experience of patient's post‐surgery, particularly in underserved contexts. It is pertinent for the multi‐disciplinary team of professionals (e.g., clinicians, psychologists, counsellors, specialist nurses, and allied health professionals) involved in the care of people with breast cancer be cognizant and trained to explore psychosocial issues and promote quality of life‐wellbeing to enhance engagement coping and vice versa in a vicious pattern. Such assessments can inform referral to support services as needed based on a shared decision process with the clients.

On a broader note, these study findings indirectly underscore the importance of expanding the implementation of psychosocial oncology services as an integral component of a holistic care, particularly in underserved settings. There is a need for stakeholders' education‐engagement, research practice, end‐users’ input, cultural sensitivity, evidence‐based planning/guidelines, training‐capacity building, and funding to support holistic care for breast cancer patients in our study setting.

In conclusion, coping strategies practiced by patients with breast cancer after surgery are multifaceted, varying based on their perception, and engagement coping was more likely in patients with improved psychosocial wellbeing‐quality of life. We advocate for interventions to bolster well‐being‐quality of life and healthy perception to promote engagement coping. Such interventions should adopt holistic model and viable strategies, involving an active integration of psychosocial services into the care of patients with breast cancer in underserved contexts. Also, there is a need for future hypothesis‐driven studies with robust methodology to guide evidence‐based implementation.

## Author Contributions

AAB, and ATO were involved in conceptualizing the research idea. AAB, JDJ‐T and ATO were vital in the data collection. AAB, JDJ‐T, PH, BL, AK, FOS and ATO were involved in the data analysis process and interpretation. AAB, PH, BL, FOS and ATO drafted the initial manuscript and AAB, BL, PH, FOS, JDJ‐T, NM, TOO, OOK, AHA, BKO, ATO provided substantial intellectual contribution in the subsequent revisions. AAB, BL, PH, FOS, JDJ‐T, NM, TOO, OOK, AHA, BKO, ATO were involved in the visualization of the current manuscript. ATO supervised the various stages of the project. All authors gave final approval of the version to be published and agreed to be accountable for all aspects of the work.

## Funding

The project did not receive any funding and no financial body was involved in the manuscript writing or data analysis.

## Ethics Statement

Ethical approval was granted by the institutional ethical committees (ethics number: BK/HP/045/P/517/VOLV/043). Written informed consent was obtained from all participants and this study adhered to the ethical standards of the institution‐national research committee and with the 1964 Helsinki Declaration and its recent amendments regarding all procedures involving human participants. The information collected was treated with strict confidentiality.

## Conflicts of Interest

The authors declare no conflicts of interest.

## Supporting information


Supporting Information S1

## Data Availability

The datasets used and analyzed during the present study are available from the corresponding author on reasonable request.
