# Supplementary material for: Coping Strategies and Perception of Patients With Breast Cancer Post‐Surgery in an Underserved Region of Nigeria
Source: Psychooncology. 2025 Dec 30;35(1):e70370. doi: 10.1002/pon.70370 (PMC12753331; doi:10.1002/pon.70370)
Supplement: Supplementary file 1 — Supporting Information S1 [file PON-35-e70370-s001.docx]

**Supplementary document 1: Relationship of engagement coping with clinico-demographic attributes and surgical perception**

|  |  |  |  |  | |  | |  | | 95% CI | | |  |
| --- | --- | --- | --- | --- | --- | --- | --- | --- | --- | --- | --- | --- | --- |
| Variables | Mean (SD) | F | t | df | p | | MD | | SE | | Lower | Upper | |
|  |  |  |  |  |  | |  | |  | |  |  | |
| *Age* |  |  |  |  |  | |  | |  | |  |  | |
| 40 years or less | 46.24 (9.92) | 0.06 | -0.34 | 70 | 0.733 | | -0.93 | | 2.70 | | -6.33 | 4.47 | |
| 41 years and above | 47.16 (9.70) |  |  |  |  | |  | |  | |  |  | |
|  |  |  |  |  |  | |  | |  | |  |  | |
| *Type of surgery* |  |  |  |  |  | |  | |  | |  |  | |
| Lumpectomy | 47.17 (11.25) | 0.02 | 0.09 | 70 | 0.931 | | 0.27 | | 3.08 | | -5.88 | 6.42 | |
| Mastectomy | 46.90 (9.46) |  |  |  |  | |  | |  | |  |  | |
|  |  |  |  |  |  | |  | |  | |  |  | |
| *Employment status* |  |  |  |  |  | |  | |  | |  |  | |
| Unemployed | 45.76 (8.93) | 1.98 | -1.83 | 70 | 0.072 | | -4.74 | | 2.60 | | -9.92 | 0.44 | |
| Employed | 50.50 (11.21) |  |  |  |  | |  | |  | |  |  | |
|  |  |  |  |  |  | |  | |  | |  |  | |
| *Complications* |  |  |  |  |  | |  | |  | |  |  | |
| Yes | 46.91 (9.85) | 0.19 | -0.108 | 70 | 0.194 | | -0.49 | | 4.52 | | -9.51 | 8.53 | |
| No | 47.40 (8.20) |  |  |  |  | |  | |  | |  |  | |
|  |  |  |  |  |  | |  | |  | |  |  | |
| Counselling |  |  |  |  |  | |  | |  | |  |  | |
| Yes | 47.47 (7.30) | 6.012 | 1.53 | 70 | 0.129 | | 3.72 | | 2.43 | | -1.12 | 8.56 | |
| No | 45.76 (8.07) |  |  |  |  | |  | |  | |  |  | |
|  |  |  |  |  |  | |  | |  | |  |  | |
| *Level of education* |  |  |  |  |  | |  | |  | |  |  | |
| Primary or less | 45.81 (7.87) | 5.58 | -1.02 | 70 | 0.311 | | -2.33 | | 2.28 | | -6.88 | 2.22 | |
| Secondary or more | 48.14 (11.30) |  |  |  |  | |  | |  | |  |  | |
|  |  |  |  |  |  | |  | |  | |  |  | |
| *Duration post-surgery* |  |  |  |  |  | |  | |  | |  |  | |
| 16 weeks or less | 46.18 (8.78) | 1.53 | -1.049 | 70 | 0. 038 | | -2.63 | | 2.522 | | -7.64 | 2.37 | |
| More than 16 weeks | 48.81 (11.63) |  |  |  |  | |  | |  | |  |  | |
| *Feelings of incompleteness* |  |  |  |  |  | |  | |  | |  |  | |
| Yes | 46.97(9.37) | 0.67 | 0..402 | 70 | 0.019 | | 3.39 | | 2.39 | | -4.68 | 4.87 | |
| No | 43.88(10.24) |  |  |  |  | |  | |  | |  |  | |
| *Satisfaction with clothing fit^b^* |  |  |  |  |  | |  | |  | |  |  | |
| Yes | 45.47(11.11) | 3.55 | -1.23 | 70 | 0.025 | | -2.79 | | 2.27 | | -7.33 | 1.75 | |
| No | 48.26(7.87) |  |  |  |  | |  | |  | |  |  | |
| *Impact on daily living* |  |  |  |  |  | |  | |  | |  |  | |
| Yes | 48.36(9.14) | 0.265 | 0.903 | 70 | 0.037 | | 2.17 | | 2.40 | | -2.62 | 6.96 | |
| No | 46.18(9.98) |  |  |  |  | |  | |  | |  |  | |

Notes: 95% CI = 95% confidence interval; df = degree of freedom; t = independent t- test; p-value = significant at p < 0.05; Mean = Mean score; SD = Standard Deviation; MD = Mean Difference; SE = Standard Error; Unemployed = Unemployed and petty trader; Counselling = Counselling before surgery. ^a^Is there a feeling of incompleteness? ^b^Are you satisfied with the way your clothes fit? ^c^Did the surgery negatively affects your house chores or daily activities

**Supplementary document 2: Relationship of disengagement coping with clinico-demographic variables and perception about surgical treatment**

|  |  |  |  |  | |  | |  | | 95% CI | | |  |
| --- | --- | --- | --- | --- | --- | --- | --- | --- | --- | --- | --- | --- | --- |
| Variables | Mean (SD) | F | t | df | p | | MD | | SE | | Lower | Upper | |
|  |  |  |  |  |  | |  | |  | |  |  | |
| *Age* |  |  |  |  |  | |  | |  | |  |  | |
| 40 years or less | 45.23 (10.87) | 0.41 | 0.71 | 70 | 0.482 | | 1.96 | | 2.78 | | -3.58 | 47.51 | |
| 41 years and above | 43.27 (9.75) |  |  |  |  | |  | |  | |  |  | |
|  |  |  |  |  |  | |  | |  | |  |  | |
| *Type of surgery* |  |  |  |  |  | |  | |  | |  |  | |
| Lumpectomy | 44.67 (8.85) | 0.91 | 0.35 | 70 | 0.726 | | 1.12 | | 3.18 | | -5.21 | 7.45 | |
| Mastectomy | 43.55 (10.25) |  |  |  |  | |  | |  | |  |  | |
|  |  |  |  |  |  | |  | |  | |  |  | |
| *Employment status* |  |  |  |  |  | |  | |  | |  |  | |
| Unemployed | 43.30 (9.30) | 0.59 | -0.65 | 70 | 0.521 | | -1.76 | | 2.73 | | -7.20 | 3.68 | |
| Employed | 45,06 (11.99) |  |  |  |  | |  | |  | |  |  | |
|  |  |  |  |  |  | |  | |  | |  |  | |
| *Complications* |  |  |  |  |  | |  | |  | |  |  | |
| Yes | 43.69 (10.15) | 0.716 | -1.53 | 70 | 0.879 | | -.713 | | 4.66 | | -10.00 | 8.58 | |
| No | 44.40 (8.26) |  |  |  |  | |  | |  | |  |  | |
|  |  |  |  |  |  | |  | |  | |  |  | |
| *Counselling* |  |  |  |  |  | |  | |  | |  |  | |
| Yes | 46.04 (11.26) | 0.084 | 1.35 | 70 | 0.181 | | 3.39 | | 2.51 | | -1.61 | 8.39 | |
| No | 42.65 (9.24) |  |  |  |  | |  | |  | |  |  | |
|  |  |  |  |  |  | |  | |  | |  |  | |
| *Education* |  |  |  |  |  | |  | |  | |  |  | |
| Primary or less | 43.65 (9.34) | 0.26 | -0.76 | 70 | 0.940 | | -0.18 | | 2.37 | | -4.91 | 4.55 | |
| Secondary or more | 43.83 (10.75) |  |  |  |  | |  | |  | |  |  | |
|  |  |  |  |  |  | |  | |  | |  |  | |
| *Duration since surgery* |  |  |  |  |  | |  | |  | |  |  | |
| 16 weeks or less | 41.91 (8.79) | 0.20 | -1.19 | 70 | 0.032 | | -5.06 | | 2.58 | | -8.21 | 2.08 | |
| More than 16 weeks | 46.84 (10.94) |  |  |  |  | |  | |  | |  |  | |
| *Feelings of incompleteness* |  |  |  |  |  | |  | |  | |  |  | |
| Yes | 46.00(9.86) | 0.19 | 2.67 | 70 | 0.009 | | 6.27 | | 2.35 | | 1.58 | 10.95 | |
| No | 39.73(9.04) |  |  |  |  | |  | |  | |  |  | |
| *Satisfaction with clothing fit^b^* |  |  |  |  |  | |  | |  | |  |  | |
| Yes | 41.03(10.21) | 3.89 | -2.24 | 70 | 0.028 | | -5.13 | | 2.29 | | -9.70 | -5.57 | |
| No | 46.16(9.23) |  |  |  |  | |  | |  | |  |  | |
| *Impact on daily living* |  |  |  |  |  | |  | |  | |  |  | |
| Yes | 46.48(8.76) | 2.24 | 1.73 | 70 | 0.089 | | 4.20 | | 2.43 | | -0.66 | 9.06 | |
| No | 42.28(10.37) |  |  |  |  | |  | |  | |  |  | |

Notes: 95% CI = 95% confidence interval; df = degree of freedom; t = independent t-test; p-value = significant at p < 0.05; Mean = Mean score; SD = Standard Deviation; MD = Mean Difference; SE = Standard Error; Unemployed = Unemployed and petty trader; Counselling = Counselling before surgery. ^a^Is there a feeling of incompleteness? ^b^Are you satisfied with the way your clothes fit? ^c^Did the surgery negatively affect your house chores or daily activities?
